# PreOBP_ML: Machine Learning Algorithms for Prediction of Optical Biosensor Parameters

**DOI:** 10.3390/mi14061174

**Published:** 2023-05-31

**Authors:** Kawsar Ahmed, Francis M. Bui, Fang-Xiang Wu

**Affiliations:** 1 Department of Electrical and Computer Engineering, University of Saskatchewan, 57 Campus Drive, Saskatoon, SK S7N 5A9, Canada; francis.bui@usask.ca; 2 Division of Biomedical Engineering, Department of Computer Science and Department of Mechanical Engineering, University of Saskatchewan, Saskatoon, SK S7N 5A9, Canada; faw341@mail.usask.ca

**Keywords:** machine learning, optical sensor, parameter estimations, prediction, performance

## Abstract

To develop standard optical biosensors, the simulation procedure takes a lot of time. For reducing that enormous amount of time and effort, machine learning might be a better solution. Effective indices, core power, total power, and effective area are the most crucial parameters for evaluating optical sensors. In this study, several machine learning (ML) approaches have been applied to predict those parameters while considering the core radius, cladding radius, pitch, analyte, and wavelength as the input vectors. We have utilized least squares (LS), LASSO, Elastic-Net (ENet), and Bayesian ridge regression (BRR) to make a comparative discussion using a balanced dataset obtained with the COMSOL Multiphysics simulation tool. Furthermore, a more extensive analysis of sensitivity, power fraction, and confinement loss is also demonstrated using the predicted and simulated data. The suggested models were also examined in terms of $R^{2}$-score, mean average error (MAE), and mean squared error (MSE), with all of the models having an $R^{2}$-score of more than 0.99, and it was also shown that optical biosensors had a design error rate of less than 3%. This research might pave the way for machine learning-based optimization approaches to be used to improve optical biosensors.

## 1. Introduction

Knight [1] first proposed the notion of photonic crystal fiber (PCF), which consists of a solid or hollow core with a microstructure arrangement extending throughout the fiber’s length. PCFs are becoming more and more widespread because of their design freedom, low cost, resilience, rapid detection, compact size, high sensitivity, and versatility. As a result of these characteristics, it is utilized in optical sensors [2,3,4], optical lasers [5,6], spectroscopy [7,8], Raman scattering [9], and a variety of other applications. The finite element method (FEM) [10,11], block-iterative frequency domain methods [12,13], and plane wave expansion methods [14,15], are all numerical approaches for improving the properties of PCFs. Such procedures, however, take a long time to complete. Furthermore, determining a PCF’s properties typically needs a considerable quantity of data, and all other parameters must be recalculated when one parameter is altered. As a result, parameter updates as well as data collection take a while to execute. Machine learning (ML) is now the most effective solution for these challenges, and it has begun to address them.

Machine learning is a technique for developing a standard function for a hypothesis or for learning specific indicators to distinguish different inputs as separate samples. A well-balanced dataset may be used to effectively train machine learning algorithms and help them in making highly accurate predictions. Over the last several years, machine learning techniques have been employed in a range of sectors, including medical research, transportation, agricultural science, plasmonics, biosensing, traffic categorization, network security, and many more. In the realm of PCF, ML has been utilized to optimize and forecast the performance of photonic crystal nanocavities [16,17,18]. To our knowledge, only a few researchers have attempted to predict optical characteristics using machine-learning approaches. An artificial neural network (ANN) model for calculating the optical characteristics of a PCF was reported by Chugh et al. [19]. They raised the number of epochs to produce a more accurate prediction as well as when the wavelength exceeds 1.5 μm, the confinement loss is dispersed. They relied only on the ANN model, with no comparisons to other machine learning approaches. Ferreira and Malheiros-Silveira proposed an ANN model based on multilayer perceptron and Extreme Learning Machine for calculating the optical characteristics of PCF in 2018 [20]. They were only concerned with PCF geometrical models. Khan et al. addressed several machine learning techniques for performance monitoring in optical communications and networking applications at the start of 2019 [21]. They opined that the TensorFlow package was useful for building machine learning algorithms. At the end of 2019 [22], Chugh et al. investigated an ML regression technique for estimating the effective index, coupling length, and power confinement in various waveguides. According to the researchers, the PyTorch and MLPRegressor models exhibit absolute percentage errors of 7–10% and 1–4%, respectively. In December 2019, the same team presented an ML technique for calculating the optical properties of a PCF [19]. They only looked at the effective refractive index (ERI) and confinement loss for up to 5000 epochs (CL). They did not display the extra design features or optical attributes that they exhibited in the previous research [19]. The usage of an ANN and a Convolutional Neural Network (CNN) in the mode classification of PCF-based surface plasmon resonance (SPR) sensor design was proposed by Khare and Goswami in 2021 [19]. They proposed a variety of methods for simply categorizing modes. However, a number of design aspects were overlooked.

When evaluating PCF design factors and forecasting optical characteristics, different ML algorithms may play an essential role in enhancing PCF quality in various aspects in a short period and with less exertion. As a consequence, machine learning methods in the field of optical sensor design and improvement show a lot of opportunities. As a result, we utilized four distinct machine learning methods that, to our information, have never been used previously in this field. Other machine-learning approaches may have been utilized in the research, but we opted for those since they are relatively new in this field. The following are the primary objectives of this study:

1. To investigate the attributes of PCF sensors using various ML algorithms.
2. To make some changes to improve the model’s accuracy as well as to minimize the error rate.
3. To estimate the output faster than direct numerical simulation strategies.

## 2. Parameter Estimation Methods

### 2.1. Machine Learning and Optimization

To make predictions or judgments without needing to be explicitly programmed to do so, ML algorithms build a model based on training data [23]. In many fields, such as health, computer vision, speech recognition, and email filtering, where constructing conventional algorithms to perform the necessary tasks is challenging or even impractical, ML methods are used [24,25]. The minimization of a loss function on a training set of instances is a common formulation for learning problems, which connects machine learning to optimization. Loss functions describe the discrepancy between the predictions of the model and the actual occurrences of the problem. These models are trained to properly predict the labels that have been assigned to a group of samples [26]. The four approaches used are briefly described below.

### 2.2. Least Squares Regression (LSR) Method

The LSR approach has traditionally been used to minimize the sum of squares of error terms with a homogeneous variance and normal distribution and, therefore, to improve the model [27]. In terms of optimization, it solves problems of the form [28]:

(1) $$\begin{array}{c} {\mathrm{Min}}_{\beta }{\left|\left|Y-X\beta \right|\right|}_{2}^{2} \end{array}$$

where *X* stands for the independent input variable, *Y* stands for the model output, and the symbol β stands for the model parameter. The LSR approach relies on a number of assumptions in order to produce trustworthy results. The regression model should be represented in a linear form, according to the starting assumption. This implies that the average error predicted by the regression model should be zero. The residual variance must be constant, and the residuals must not be correlated with one another (no autocorrelation) [29]. Certain assumptions must be made while using the LSR technique, such as the absence of a linear relationship between independent variables. The model reliability is contingent on the LSR assumptions being met. When all of these conditions are met, the LSR approach can produce the best results. Multicollinearity is defined as a linear relationship between the independent variables. While multicollinearity improves the covariance and variance of regression coefficients, none or only a few of the explanatory variables will be statistically significant if the model’s R${}^{2}$ value is changed. As a result, model misunderstanding is caused by multicollinearity [30].

The LSR method has a significant benefit over all other estimating methods, such as traverse adjustments, in which it is theoretically and statistically defensible and hence a fully rigorous procedure. By minimizing the sum of the squares of the mistakes to a minimal value, the LSR principle states that it is possible to determine the most likely values of a system of unidentified variables on which measurements have been made.

### 2.3. LASSO Method

For linear modeling, LASSO stands for Least Absolute Shrinkage and Selection Operator. In linear models, the LASSO regression method is a regularization technique for estimating unknown parameters [31]. It modifies model parameters based on a loss function while taking model complexity into account. When there are significant correlations between predictors, the LASSO will randomly select one and ignore the others, and when all predictor variables are comparable, it will collapse [32]. The LASSO penalty forces the majority of coefficients to be zero, with only a small percentage likely to be nonzero. It usually consists of a linear model with an additional regularization parameter. The following is the objective function [33] that must be minimized

(2) $$\begin{array}{c} {\mathrm{Min}}_{\beta }\frac{1}{2n_{\mathrm{samples}}}{\left|\left|Y-X\beta \right|\right|}_{2}^{2}+\lambda {\left|\left|\beta \right|\right|}_{1} \end{array}$$

where λ≥ 0 signifies the regularization constant and ${\left|\left|\beta \right|\right|}_{1}$ denotes the coefficient vector’s $\ell_{1}$-norm penalty. For correctly selected λ, the $\ell_{1}$ penalty allows the LASSO to regularize the least squares fit while simultaneously decreasing certain components of β to zero. The cyclical coordinate descent [32] method is faster than the LRS method [28] at evaluating all LASSO solution routes for λ. The LASSO is a common feature selection approach due to these factors. Nonetheless, the LASSO has three major flaws: it lacks the oracle feature, is incompatible with high-dimensional data, and cannot select more features than the sample size without saturating [34]. On the other hand, because the coefficients have been decreased and deleted, it can deliver exact predictions without a large increase in biases.

### 2.4. Elastic-Net (ENet) Method

The ENet approach is a LASSO extension that can handle features with strong correlations [32]. The ENet was suggested to analyze high-dimensional data in order to avoid the instability of LASSO when predictors were significantly linked [34]. The ENet approach combines $\ell_{1}$ (Lasso) and $\ell_{2}$ (Ridge Regression) penalties, and it can be represented mathematically as [28]:

(3) $$\begin{array}{c} {\mathrm{Min}}_{\beta }\frac{1}{2n_{\mathrm{samples}}}{\left|\left|Y-X\beta \right|\right|}_{2}^{2}+\lambda \rho {\left|\left|\beta \right|\right|}_{1}+\frac{\lambda (1-\rho )}{2}{\left|\left|\beta \right|\right|}_{2}^{2} \end{array}$$

The regularization constant ρ is $\ell_{1}$ ratio, and the $\ell_{1}$ and $\ell_{2}$ norm penalties of regularization coefficients are ${\left|\left|\beta \right|\right|}_{1}$ and ${\left|\left|\beta \right|\right|}_{2}^{2}$. When numerous properties are linked together, the ENet is effective. The ENet’s $\ell_{1}$ section automatically chooses variables, whereas the $\ell_{2}$ segment allows for grouped selection and regulates solution paths to improve prediction. The ENet may discover and choose groups of correlated variables even when the groupings are unknown by using a grouping effect throughout the variable selection, such that a group of very correlated features appears to have coefficients of the same scale. When p≫n, where *n* is the sample size and *p* is the predictor numbers, the ENet chooses more than *n* variables, unlike the LASSO. The ENet, on the other hand, lacks the oracle property.

### 2.5. Bayesian Ridge Regression (BRR) Method

BRR is a subset of Bayesian regression that belongs to the ridge regression category, and it possesses all of the properties of both Bayesian and ridge regression [35]. The Gaussian distribution is used in Bayesian regression, which is a probabilistic method [36]. In order to optimize posterior prediction, the $\ell_{1}$ regularization must be used. The weighted coefficient *W* is determined from a Gaussian distribution, which is the only difference between Bayesian and BRR. The Gaussian prior considerably reduces the magnitude of all effects in BRR. A spherical Gaussian provides the prior *W* for the coefficient:

(4) $$P\left(W\right|\lambda )=\left(W\right|0,\lambda^{-1}I_{P})$$

The priors over α and λ are gamma distributions, which are the conjugate prior for Gaussian precision. By default, the value of $\lambda_{1}$, $\lambda_{2}$, $\alpha_{1}$, and $\alpha_{2}$ is $10^{-6}$. BRR is the name given to the resulting model, which is comparable to the classic Ridge. $I_{P}$ represents the identity matrix. Although BRR takes a long time to run, it excels at adjusting to small data parameters and is simple to use in regularization problems and change hyper-parameters. Furthermore, the BRR focuses on feature selection in order to limit the number of inputs and then rank them according to their predictive value to the estimation method. In reality, BRR produces similar results to the maximum a posterior (MAP) technique.

## 3. Optical Sensor Numerical Models

### 3.1. Optical Sensor

Due to their great precision and sensitivity, optical sensors are becoming increasingly popular. According to their design and measurement procedures, the most common optical sensors can be grouped into three categories: (1) spectroscopy-based sensors, (2) SPR-based sensors, and (3) negative curvature-based sensors. Fiber optical sensors have been developed for various applications such as environmental monitoring, salinity monitoring, temperature monitoring, magnetic fluid monitoring, agricultural monitoring, industrial monitoring, food quality monitoring, health monitoring, and military operations over the last two decades in medicine, air force, and industry. Optical biosensors, among many uses of optical sensors, usher in a new age in the field of biological monitoring and detection [3,37].

Optical biosensors are analytical smart devices that are used to monitor a variety of biological components in conjunction with the appearance of chemicals or analytes. Nowadays, PCF-based biosensors are utilized to detect physiological components such as RBCs, hemoglobin, plasma cells, WBCs, cancer cells, tuberculosis, pregnancy, urine, medications, virus, SARS-CoV-2, and so on. The large variety of real-time applications of optical biosensors, as well as design freedom, fast detection time, compact size, simplicity, resilience, and low cost, have piqued researchers’ interest. As a result, in recent years, a number of researchers have looked at using plasmonic materials rather than gold and silver to improve the quality of optical biosensors. ML techniques are also used to speed up the detection process and improve accuracy. This quick biochemical diagnosis will help to reduce the danger of contamination and exposure [38,39,40,41].

**Figure 1:**
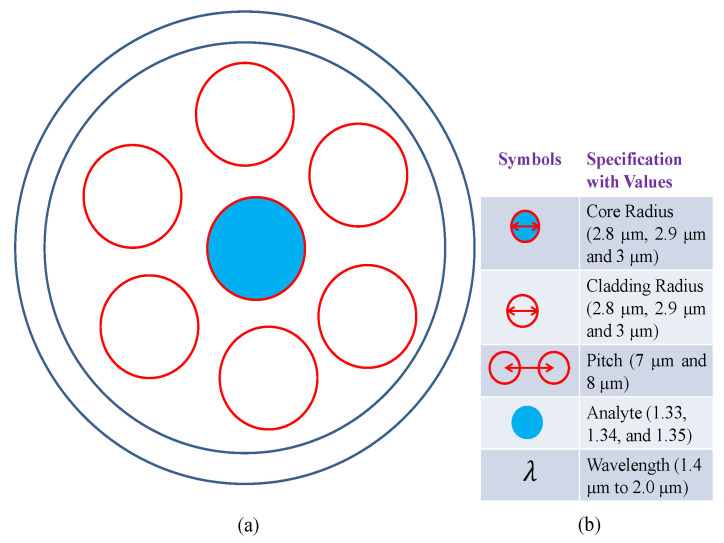
(**a**) Cross-sectional image of the proposed optical biosensor; (**b**) Design-dependent parameters or specifications.

Figure 1 shows the cross-sectional illustration of an optical biosensor with specifications. Core radius, cladding radius, pitch, analyte, and wavelength are five optical design factors for the proposed biosensor. The radius of the core and cladding in this study ranged from 2.8 µm to 3.00 µm, with a pitch of between 7.00 µm and 8.00 µm. Furthermore, during the operating range of wavelength 1.4 µm to 2.0 µm, the examined analyte range was 1.33 to 1.35. Some of the most important optical guiding properties are described as follows.

### 3.2. Effective Refractive Index (ERI)

ERI is a number that compares the phase delay in a waveguide per unit length to the phase delay in a vacuum. ERI is symbolized as $n_{\mathrm{eff}}$, with RIU as the unit. ERI can be expressed mathematically as follows:

(5) $$\begin{array}{c} n_{\mathrm{eff}}=A\pm iB \end{array}$$

where *A* stands for the real value of ERI and *B* stands for an imaginary value of ERI.

### 3.3. Optical Power Profiles (OPP)

Optical sensors are used to monitor buildings that originate, supply, distribute, and transform electrical power in the energy field. Core power, cladding power, and total power are the three categories of optical sensor energies. An optical sensor’s performance is ensured by its high core power.

### 3.4. Optical Power Fraction (OPF)

The OPF is a unitless quantity that specifies how much light enters the optical sensor. Core and cladding optical power fractions are two different forms of optical power fractions. Core power fractions (CPF) with high values suggest the highest sensitivity and lowest cladding power fraction (CLPF). Mathematically, the total, core, and cladding powers are computed as follows [3,37,38,39]:

(6) $$\begin{array}{c} \mathrm{Total}\;\mathrm{Power}=\int_{total}Re\left(E_{x}H_{y}-E_{y}H_{x}\right)dx\;dy \end{array}$$

(7) $$\begin{array}{c} \mathrm{Core}\;\mathrm{Power}=\int_{Core}Re\left(E_{x}H_{y}-E_{y}H_{x}\right)dx\;dy \end{array}$$

(8) $$\begin{array}{c} \mathrm{Clading}\;\mathrm{Power}=\int_{Cladding}Re\left(E_{x}H_{y}-E_{y}H_{x}\right)dx\;dy \end{array}$$

where the transverse electric and magnetic fields are denoted by the symbols $E_{x}$, $E_{y}$, $H_{x}$, and $H_{y}$, respectively. Mathematically, the core and cladding power fractions are computed as follows [3,37,38,39]:

(9) $$\begin{array}{c} \mathrm{CPF}\left(\%\right)=\frac{\mathrm{Core}\;\mathrm{Power}}{\mathrm{Total}\;\mathrm{Power}}\times 100 \end{array}$$

(10) $$\begin{array}{c} \mathrm{CLPF}\left(\%\right)=\frac{\mathrm{Cladding}\;\mathrm{Power}}{\mathrm{Total}\;\mathrm{Power}}\times 100 \end{array}$$

### 3.5. Optical Effective Area (OEA)

The area that a waveguide or fiber mode successfully covers in the transverse dimensions is measured in this mode of operation. Fiber or other waveguide modes have smooth transverse profiles, making defining a mode area difficult, especially for sophisticated mode shapes where a $\frac{1}{e^{2}}$ intensity requirement, such as for Gaussian beams, is not appropriate. OEA is symbolized as $A_{\mathrm{eff}}$, with m${}^{2}$ as the unit. The OEA can be defined as follows [3,37,38,39]:

(11) $$\begin{array}{c} A_{\mathrm{eff}}\left(m^{2}\right)=\frac{{\left(\int \int |E\right|}^{2}{dx\;dy)}^{2}}{\left(\int \int |E{|}^{4}dx\;dy\right)} \end{array}$$

where *E* is the amplitude of the electric field.

### 3.6. Optical Loss Profiles (OLP)

To fulfill the boundary condition, a circular perfectly matched layer (PML) is utilized, which prevents possible reflection at the border. The imaginary component of the ERI can be used to calculate confinement or leakage loss. OLP is symbolized as $L_{c}$, with dB/cm as the unit. The confinement or leakage loss is calculated as follows [3,37,38,39]:

(12) Lc(dB/cm)=8.686×2πλ×Im(neff)×104

In this case, the wave number in free space is $\frac{2\pi }{\lambda }$. $L_{c}$ is the result of scattering light escaping from the core to the outer materials, which is calculated using the imaginary portion of $n_{\mathrm{eff}}$ at the specific wavelength λ.

### 3.7. Optical Sensing Profile (OSP)

The relative sensitivity coefficient measures the interaction between light and the analyte, which can be calculated as follows [3,37,38,39].

(13) $$\begin{array}{c} \mathrm{Sensitivity}\left(\%\right)=\frac{n_{r}}{n_{\mathrm{eff}}}\times \mathrm{CPF} \end{array}$$

where $n_{r}$ denotes the refractive index of the detected liquid within the core and $n_{\mathrm{eff}}$ denotes the model effective index.

### 3.8. Limitations and Proposed Solutions

Due to restrictions, any changes in optical sensor design parameters will result in changes in sensor performance, which is time-consuming to optimize. Different ML parameter estimate models that are faster and more accurate can be used to solve the optical sensor design optimization challenge.

## 4. Methodology

**Figure 2:**
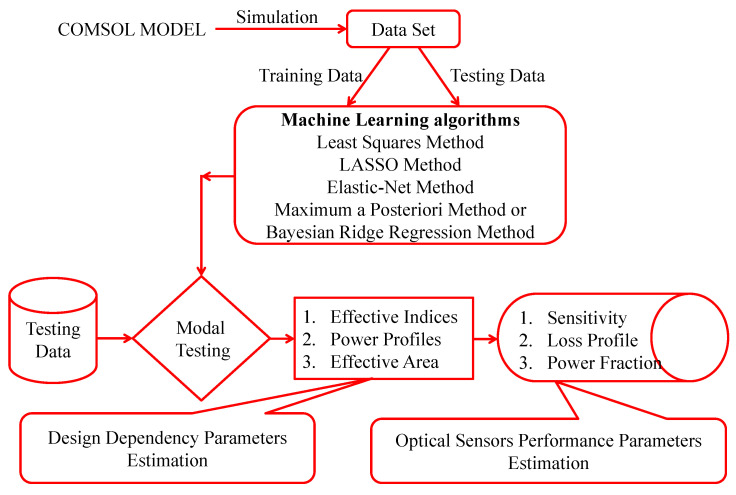
Flowchart of the research’s procedure. First, the design dependency parameters are estimated, and then the optical Sensors’ Performance Parameters are calculated.

For the proposed research, quantitative data is necessary. The data was gathered through simulation. After some preparation, such as handling noisy data and measuring the correlation between variables using Pearson’s correlation testing method, a valid dataset was formed. In order to determine the best one, regression techniques are applied. Figure 2 illustrates the full procedure of the research.

### 4.1. Design and Dataset Collection

Data collection is a methodical process for gathering and analyzing particular information in order to respond to pertinent questions and evaluate results. It focuses on discovering all there is to know about a certain subject. Data is obtained in an effort to test hypotheses in an effort to comprehend a phenomenon. Here was created a rudimentary PCF model, which is shown in Figure 1.

### 4.2. Dataset Distribution

A technique or list that shows all potential values (or intervals) is a distribution of the data. Additionally, it displays the frequency of each value, which is important. Figures make it simple to evaluate both the numbers and the frequency in which they exist. A project’s data is typically organized from the smallest to the largest. The dataset was split into training and testing portions by 70% and 30%, respectively, in this research.

### 4.3. Training, Testing and Evaluation

The dataset was fed into the ML algorithms for training, and the model was evaluated using a new tuple. In this research, 690 data points from 1402 were utilized to train the models, and 13 data points were used to visualize the model performance.

## 5. Result Analysis and Discussion

The trained models have been validated in this section. Unknown input parameters have been used to test the model. With wavelength changes ranging from 1.4 µm to 2.0 µm, the suggested model has been trained to predict effective indices, core power, and total power. After that, sensitivity, confinement loss, core power percentage, and effective mode area are calculated. This section includes various graphical visualizations and comparison tables in terms of the $R^{2}$-score, MSE, and MAE.

### 5.1. ERI for X-Axis and Y-Axis

**Figure 3:**
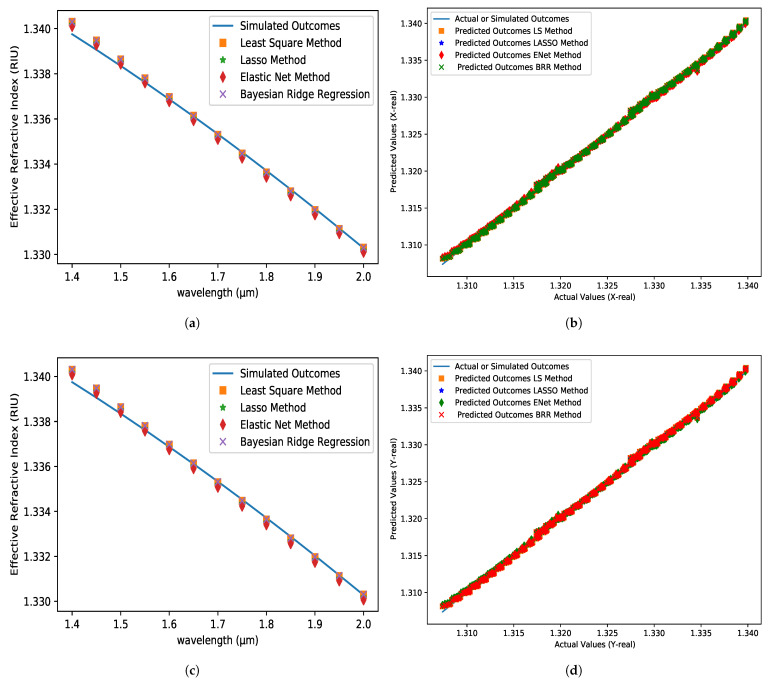
The real value of the effective refractive index (RIU) variations with respect to the wavelength at (**a**) X-axis and (**c**) Y-axis for analyte 1.35, pitch = 8 µm, core and cladding radius = 3 µm. The comparison between the predicted dataset (tuning) for different algorithms and the actual dataset obtained from FEM simulation at (**b**) X-axis and (**d**) Y-axis.

**Figure 4:**
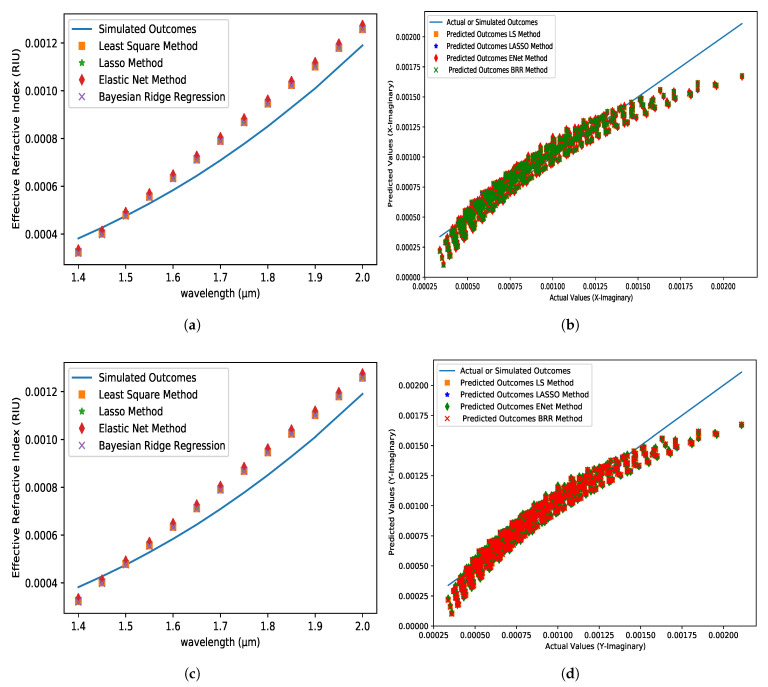
The imaginary value of ERI variations with respect to the wavelength at (**a**) X-axis and (**c**) Y-axis for analyte 1.35, pitch = 8 µm, core and cladding radius = 3 µm. The Comparison between the predicted dataset (tuning) for different algorithms and the actual dataset obtained from FEM simulation at (**b**) X-axis and (**d**) Y-axis.

The predicted and simulated outcome values are represented by a straight line and different symbols in Figure 3a,c for real values of ERI in the X-axis and Y-axis, respectively. Methods are represented by different color dots, and the solid line represents the simulated values. The ERI values drop as the wavelength rises. The predicted values in Figure 3a,c are substantially equal to the simulated values. The greatest value of $R^{2}$ for both the X-axis and the Y-axis is 0.9994 using the LS and BRR methods. However, when compared to other applied techniques, the LS method has the lowest mean square error (MSE) of $3.9729\times 10^{-8}$ and $3.9921\times 10^{-8}$ for both the X-axis and Y-axis, respectively. Furthermore, all applied approaches have an MAE of 0.0001 in common. Table 1 shows the similarities in greater detail. All of the algorithms predict results that are nearly indistinguishable in the imaginary section of the ERI. At first, the predicted values are lower than the simulated values, but after 1.5 µm, the predicted values are higher than the simulated values in Figure 4a,c. The LS method has the highest $R^{2}$ of 0.9436 and 0.9435, the lowest MSE of $5.7626\times 10^{-9}$ and $5.7656\times 10^{-9}$, and the lowest MAE of $6.0969\times 10^{-5}$ and $6.1013\times 10^{-5}$ for the X-axis and Y-axis, respectively, when compared to all other strategies. Table 2 shows the results of the detailed comparisons and also exhibits that the ENet method has the weakest performance when compared to the other strategies. However, the ENet approach had an absolute error of 0.00015 at 1.85 µm wavelength, which is fantastic news. As a result, we may state that practically all used approaches have a 1–5 percent error rate. Furthermore, the results of the proposed sensors in both axes indicate similar results, which is why the fundamental mode is chosen for further investigation.

### 5.2. Effective Mode Area (EMA)

**Figure 5:**
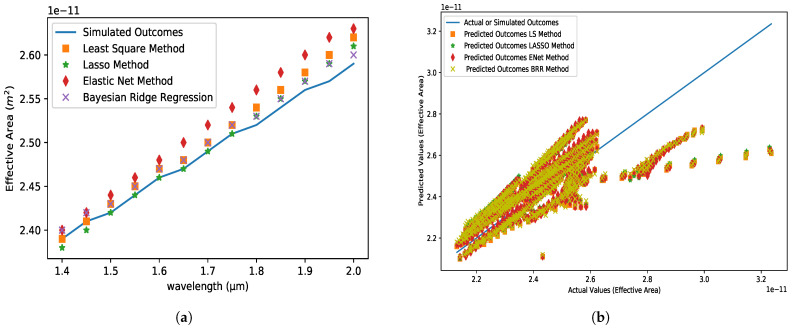
(**a**) Effective area variations with respect to wavelength for analyte 1.35, pitch = 8 µm, core and cladding radius = 3 µm and (**b**) the comparison between the predicted dataset for different algorithms and actual dataset obtained from FEM simulation.

Figure 5 depicts the EMA as a function of wavelength variations over the working range of 1.4 to 2.0 µm, which clearly shows that there is no noticeable difference between the estimated model results and the simulated outcomes. In comparison to the four applied models, it is also obvious that LS approaches perform the best and ENet performs the poorest. The absolute inaccuracy for the ENet approach is $3.1\times 10^{-13}$ at 1.8 µm wavelength, as seen in Figure 5. It demonstrates that the differences between predicted and simulated results are nearly identical. Furthermore, the MSE of least square, LASSO methods is of the order of $10^{-25}$, whereas ENet, BRR methods are of the order of $10^{-24}$. Furthermore, the MAE of all applicable methods is of the order of $10^{-13}$.

### 5.3. Total Power and Core Power

**Figure 6:**
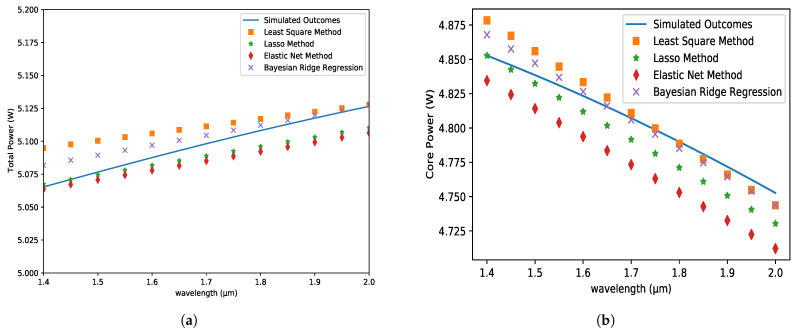
(**a**) Total Power and (**b**) Core Power variations with respect to the wavelength at the X-axis for analyte 1.35, pitch = 8 µm, core and cladding radius = 3 µm.

Core power and total power fluctuate due to light intensity in the core and structural changes in the fiber. The total power values of the PCF model grow as the wavelength increases in Figure 6a. As the wavelength gets longer, the total power values for the least squares and maximum posterior approaches get closer to the simulated one. In contrast, the LASSO and ENet approaches represent the reversed scenario. For lower wavelength values, the expected values are virtually comparable. Figure 6b shows that the ENet and LASSO methods produce lower values than the simulated ones, but the LS and BRR methods produce higher values for wavelengths between 1.4 µm and 1.8 µm, and then the values approach the simulated values.

### 5.4. Core Power Fraction (CPF)

**Figure 7:**
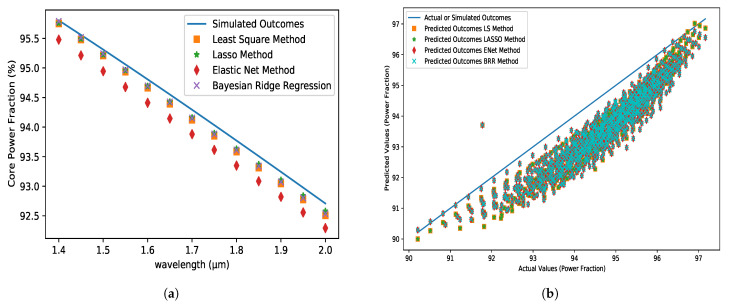
(**a**) CPF (%) variations with respect to the wavelength at the X-axis for analyte 1.35, pitch = 8 µm, core and cladding radius = 3 µm and (**b**) the comparison between the predicted dataset for different algorithms and actual dataset obtained from FEM simulation.

A power fraction is needed to calculate sensitivity. It refers to the ratio of total power to core power. The suggested PCF model has a lower power percentage for longer wavelengths. Figure 7a shows that all of the approaches had lower values than the simulated ones. Furthermore, the LS, LASSO, and BRR techniques predict closer outcomes at lower wavelengths and show absolute errors on the order of $10^{-5}$ at the lower operating wavelength (1.45 µm), respectively, while ENet displays $10^{-2}$. At the upper working point of wavelength (1.90 µm), the LS, LASSO, and BRR approaches show an absolute error of the order of $10^{-3}$, while the ENet method displays $10^{-2}$.

### 5.5. Confinement Loss Profile (CLP)

**Figure 8:**
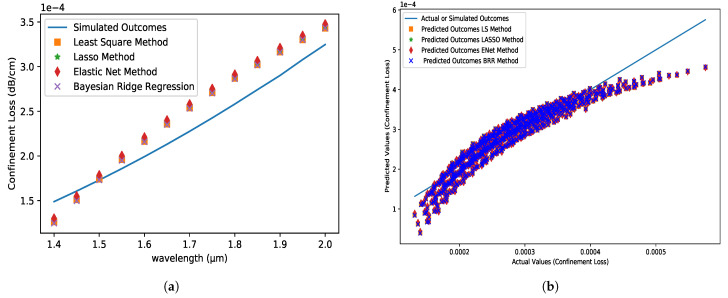
(**a**) CL (dB/cm) profile variations with respect to the wavelength at the X-axis for analyte 1.35, pitch = 8 µm, core and cladding radius = 3 µm and (**b**) the comparison between the predicted dataset for different algorithms and actual dataset obtained from FEM simulation.

CL is a critical feature of PCF, and the mode’s leaky nature causes it. The wavelength and the imaginary component of the effective index of the core-guided mode determine it. The CL is increasing in response to the increase in wavelength. The CL of the proposed model is shown to be very small in this research. The CL values for all of the techniques in Figure 8a are the same, with predicted values smaller than simulated values in the range of 1.4 µm to 1.5 µm. All of the other values are greater than the simulated values. When all approaches are used, the absolute inaccuracy is on the order of $10^{-5}$.

### 5.6. Optical Sensitivity Profile (OSP)

**Figure 9:**
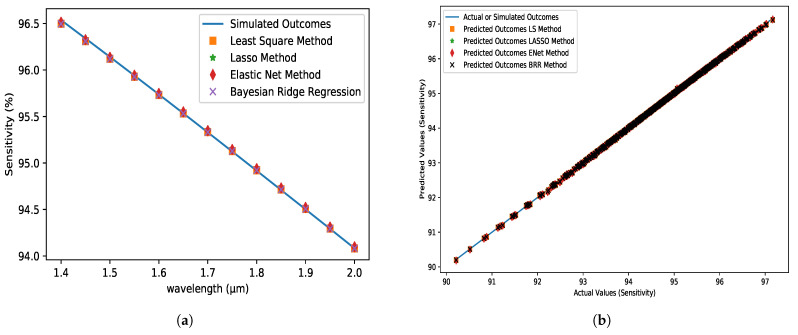
(**a**) Sensitivity (%) variations with respect to the wavelength at the X-axis for analyte 1.35, pitch = 8 µm, core and cladding radius = 3 µm. (**b**) The Comparison between the predicted dataset for different algorithms and the actual dataset obtained from FEM simulation.

The power fraction, analyte, and ERI all influence PCF sensitivity. The predicted values of various ML models, as well as simulated values, were used. The suggested PCF model has a higher sensitivity for shorter wavelengths. As demonstrated in Figure 9a, all of the predicted values are identical to the simulated values. For assessing the sensitivity profile of the proposed optical sensor, almost all approaches function well, with absolute errors in the order of $10^{-8}$. Table 3 demonstrates that the LS method outperforms other strategies, with an $R^{2}$ of 0.9994, an MSE of $3.9\times 10^{-8}$, and an MAE of $1.5\times 10^{-4}$. The ENet approach, on the other hand, has the lowest performance, with an $R^{2}$ of 0.9990, an MSE of $6.2\times 10^{-8}$, and an MAE of $1.9\times 10^{-4}$.

### 5.7. OSP Evaluation for Different Volume of Datasets

**Figure 10:**
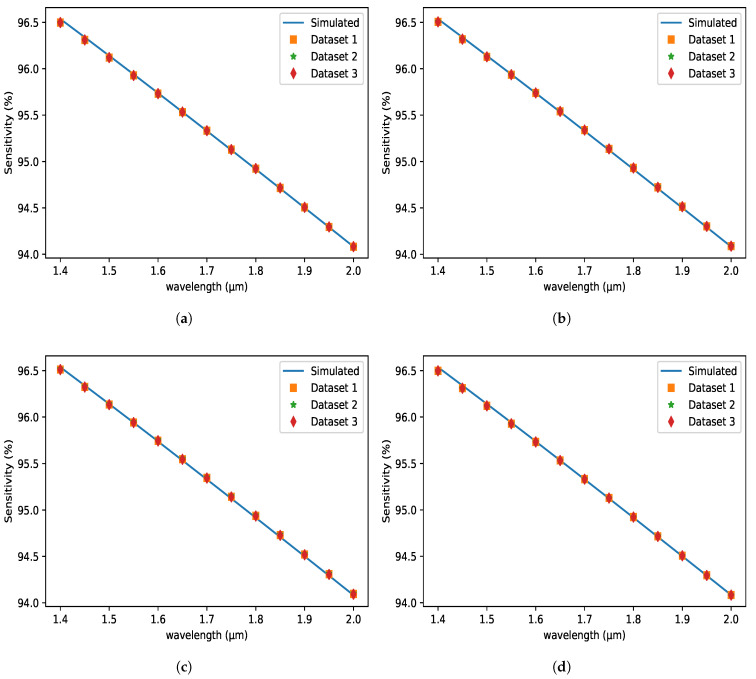
Sensitivity (%) variations with respect to the wavelength at the X-axis for different datasets using (**a**) LS (**b**) LASSO (**c**) ENet (**d**) BRR Methods.

The performance of different methods for the proposed sensor and various numbers of input datasets is shown in Figure 10. It is obvious from the illustration that the performance of applied algorithms improves as the amount of trained data grows. In comparison to dataset-1, datasets 2 and 3 include 1.5 and 2 times more data. It is now clearly demonstrated that when the volume of the dataset grows, the performance of the applied methods for the presented sensors improves as well. The changes in the approaches used are, however, relatively minor. The various performance indicator values of the employed methods are shown in Table 4. Finally, we can state that the applied ML algorithms exhibit remarkably consistent performance across a wide range of trained datasets.

### 5.8. OSP Evaluation for Different Volume of Outliers

**Figure 11:**
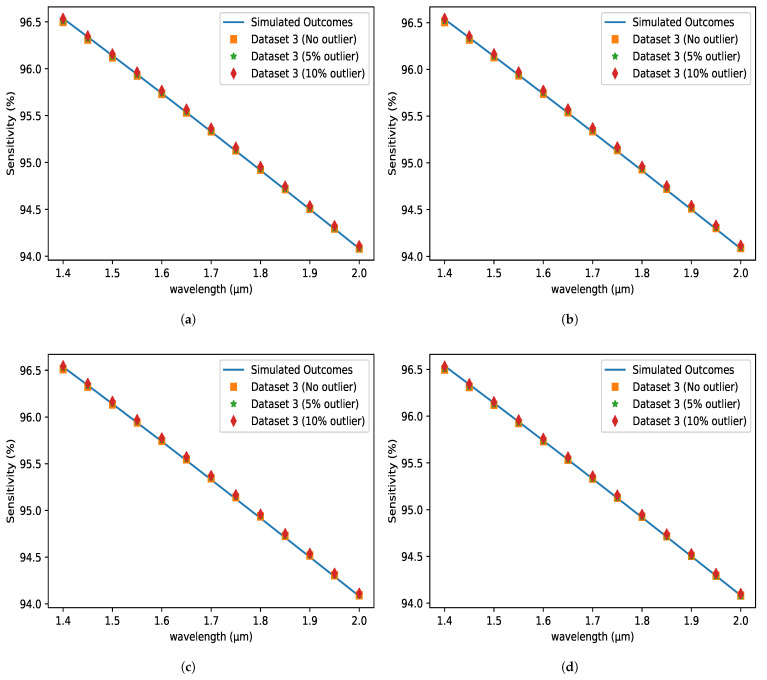
Sensitivity (%) variations with respect to the wavelength at the X-axis for different outliers using (**a**) LS (**b**) LASSO (**c**) ENet (**d**) BRR Methods.

The sensitivity performance of several approaches for various volumes of outliers is depicted in Figure 11. It is obvious from the preceding study that the applied approaches produce stable results for the input data when outliers are absent. To test the performance of the applied methods, 5% and 10% outliers are introduced to the input data, and the methods are trained repeatedly. In comparison to Dataset-3, Datasets 4 and 5 include 5% and 10% outliers, respectively. The provided approaches then demonstrated stable performance for the proposed sensor with outlier data as well. Table 5 depicts the changes in technique performance by displaying several performance indicator values. The performance of different applied procedures, however, varies slightly. Even so, the effectiveness of the LS method is consistent across diverse groups of outliers.

### 5.9. Overall Performance Evaluation

As a consequence, the LS method clearly outperforms the other four techniques when it comes to predicting outcomes. Because the trained data was properly distributed with no outliers, the LS offers the best results. So, we also put the performance of the ML techniques to the test by merging outliers ranging from 5% to 10% of the total data. We noticed that LS $R^{2}$-score performance was decreasing, from 99.94 percent to 96.52 percent, while other techniques did not degrade as significantly. After training the model, fast and precise prediction of PCF parameters is possible in a few milliseconds, which is approximately 99 percent faster than standard numerical simulation approaches. Finally, for optimizing an optical biosensor design, the proposed LS technique is the most effective when high-quality training data is available.

## 6. Conclusions

We proposed four alternative machine learning algorithms in this study to predict optical sensor design-dependent characteristics as well as optical sensor attributes. By adjusting the core and cladding radius (2.8 µm to 3 µm), pitches (7 µm and 8 µm), analytes (1.33 to 1.35) and wavelengths (1.4 µm to 2.00 µm), the mode effective indices, effective area, core, and total power confinement of suggested optical biosensors are obtained. A total of 690 data points are utilized for training the proposed ML model, with another 13 data points used to visualize the trained model performance. Almost all machine learning algorithms effectively anticipate optical design-dependent parameters and optical sensor qualities (about 99.94%), which are close to simulated values. The best LS technique achieves the highest $R^{2}$-score of 0.9994, the lowest MAE of $3.9\times 10^{-8}$, and the MSE of $1.5\times 10^{-4}$ for sensitivity profile prediction. On the other hand, the worst Elastic-Net approach has an $R^{2}$-score of 0.9990, an MAE of $6.2\times 10^{-8}$, and an MSE of $1.9\times 10^{-4}$. As a result, it is evident that the least squares method outperforms the other three techniques in terms of prediction outcomes. We also test the performance of the ML approaches by combining outliers ranging from 5% to 10% of the total data. We found that the $R^{2}$-score performance of LS fell from 99.94 percent to 96.52 percent. Finally, the proposed LS approach is efficient for optimizing an optical biosensor design, assuming that the model parameters are consistent and that training data conform to the true distribution without outliers.

## Figures and Tables

**Table 1:** Comparisonsof different applied methods for estimating the real values of effective refractive index (RIU) when analyte 1.35, pitch = 8 µm, core and cladding radius = 3 µm.

Applied Methods		X-Axis			Y-Axis	
	$R^{2}$	$\mathrm{MSE}\times 10^{-8}$	MAE	$R^{2}$	$\mathrm{MSE}\times 10^{-8}$	MAE
Least Squares	0.9994	3.9729	0.0001	0.9994	3.9721	0.0001
LASSO	0.9993	4.5486	0.0001	0.9993	4.5581	0.0001
Elastic-Net	0.9991	6.1648	0.0001	0.9992	5.8462	0.0001
B. Ridge Regression	0.9994	4.0301	0.0001	0.9994	4.5095	0.0001

**Table 2:** Comparisons of different applied methods for estimating the imaginary values of effective refractive index (RIU) when analyte 1.35, pitch = 8 µm, core and cladding radius = 3 µm.

Applied Methods		X-Axis			Y-Axis	
	$R^{2}$	$\mathrm{MSE}\times 10^{-9}$	$\mathrm{MAE}\times 10^{-5}$	$R^{2}$	$\mathrm{MSE}\times 10^{-8}$	$\mathrm{MAE}\times 10^{-5}$
Least Squares	0.9436	5.7626	6.0969	0.9435	5.7626	6.1013
LASSO	0.9421	5.9223	6.1785	0.9420	5.9274	6.1804
Elastic-Net	0.9318	6.0952	6.2524	0.9318	6.0964	6.2516
B. Ridge Regression	0.9319	8.3652	7.0367	0.9319	8.3645	7.0382

**Table 3:** Comparison of performance evaluation of different Parameter Estimation Methods for sensitivity profile.

Applied Methods	$R^{2}$	$\mathrm{MSE}\times 10^{-8}$	$\mathrm{MAE}\times 10^{-4}$
Least Squares Method	0.9994	3.90	1.50
LASSO Method	0.9993	4.50	1.60
Elastic-Net Method	0.9990	6.20	1.90
Bayesian Ridge Regression Method	0.9994	4.00	1.90

**Table 4:** Comparison of performance evaluation of different Parameters Estimation Methods for different volumes of the dataset.

Applied Methods	Dataset	$R^{2}$	$\mathrm{MSE}\times 10^{-8}$	$\mathrm{MAE}\times 10^{-4}$
Least Squares Method	Dataset-1	0.9994	3.97	1.51
"	Dataset-2	0.9993	3.86	1.50
"	Dataset-3	0.9995	3.66	1.56
LASSO Method	Dataset-1	0.9993	4.55	1.58
"	Dataset-2	0.9994	4.41	1.66
"	Dataset-3	0.9994	4.63	1.61
Elastic-Net Method	Dataset-1	0.9991	6.16	1.91
"	Dataset-2	0.9991	6.30	2.01
"	Dataset-3	0.9993	5.82	1.82
Bayesian Ridge Regression Method	Dataset-1	0.9994	4.03	1.52
"	Dataset-2	0.9994	3.95	1.56
"	Dataset-3	0.9994	4.57	1.60

**Table 5:** Comparison of performance evaluation of different Parameter Estimation Methods for different volumes of outliers.

Applied Methods	Dataset	$R^{2}$	MSE	MAE
Least Squares Method	Dataset-3	0.9995	$3.66\times 10^{-8}$	$1.56\times 10^{-4}$
"	Dataset-4	0.9897	$8.29\times 10^{-7}$	$3.05\times 10^{-4}$
"	Dataset-5	0.9657	$2.73\times 10^{-6}$	$5.84\times 10^{-4}$
LASSO Method	Dataset-3	0.9994	$4.63\times 10^{-8}$	$1.61\times 10^{-4}$
"	Dataset-4	0.9893	$8.55\times 10^{-7}$	$3.04\times 10^{-4}$
"	Dataset-5	0.9655	$2.76\times 10^{-6}$	$6.00\times 10^{-4}$
Elastic-Net Method	Dataset-3	0.9993	$5.82\times 10^{-8}$	$1.82\times 10^{-4}$
"	Dataset-4	0.9890	$8.81\times 10^{-7}$	$3.20\times 10^{-4}$
"	Dataset-5	0.9393	$5.02\times 10^{-6}$	$6.01\times 10^{-4}$
Bayesian Ridge Regression Method	Dataset-3	0.9994	$4.57\times 10^{-8}$	$1.60\times 10^{-4}$
"	Dataset-4	0.9854	$1.20\times 10^{-6}$	$3.47\times 10^{-4}$
"	Dataset-5	0.9395	$5.01\times 10^{-6}$	$5.74\times 10^{-4}$

## Data Availability

The dataset is available upon request to the corresponding author.
